# Note from the editors: World Health Organization declares novel coronavirus (2019-nCoV) sixth public health emergency of international concern

**DOI:** 10.2807/1560-7917.ES.2020.25.5.200131e

**Published:** 2020-02-06

**Authors:** Eurosurveillance editorial team

**Affiliations:** 1 European Centre for Disease Prevention and Control (ECDC), Stockholm, Sweden

Yesterday, on 30 January 2020, the World Health Organization (WHO) declared the emergence of the novel coronavirus (2019-nCoV) a public health emergency of international concern (PHEIC). This decision by WHO Director-General (DG) Tedros Adhanom Ghebreyesus, was made following the recommendations of the Emergency Committee in its second meeting [1] convened under the International Health Regulations (IHR) (2005) [2]. In his decision, the WHO DG stressed ‘*the potential for the virus to spread to countries with weaker health systems, and which are ill-prepared to deal with it*’ as one major concern and the importance of ‘*preventing the spread of the virus and ensuring a measured and evidence-based response’* [3]*.*

This sixth PHEIC under the IHR follows those declared in 2009 for the H1N1 influenza pandemic, in 2014 for the international spread of poliovirus and the Ebola outbreak in West Africa, in 2016 for the 2015/16 Zika virus epidemic and in July 2019 for the Kivu Ebola epidemic.

Following the declaration of the PHEIC, we would like to remind the readers of *Eurosurveillance* about our dedicated ‘2019 novel coronavirus collection’ that we update regularly with new articles. It already contains several articles about, for example, the development of a diagnostic methodology based on RT-PCR without the need for virus material, the severity among hospitalised cases, early-stage estimates of the importation risk to Europe, and estimated patterns of early human-to-human transmission.

The European Centre for Disease Prevention and Control (ECDC) provides regularly updated information relevant to Europe through on a dedicated webpage. Besides general information including Q&As, daily case counts, and maps with disease distribution, examples of latest updates comprise: Algorithm for management of contacts of probable or confirmed 2019-nCoV cases, Advice to healthcare workers: management of patients with 2019-nCoV infection and Public health management of persons having had contact with novel coronavirus cases in the European Union. The ECDC also publishes regular risk assessments, the Box contains the summary from the one published today.

Box. **Summary of the ECDC rapid risk assessment from 31 January 2020**

On 31 December 2019, a cluster of pneumonia cases of unknown aetiology was reported in Wuhan, Hubei Province, China. On 9 January 2020, China CDC reported a novel coronavirus (2019-nCoV) as the causative agent of this outbreak, which is phylogenetically in the SARS-CoV clade.

As of 30 January 2020 09:00, more than 7 000 laboratory-confirmed 2019-nCoV cases have been reported worldwide, mainly in China, but also with more than 70 imported cases from other countries around the world. Details on the epidemiological update for 2019-nCoV can be found on ECDC’s website.

So far, one hundred and seventy deaths associated with this virus have been reported. On 20 January, Chinese health authorities confirmed human-to-human transmission outside of Hubei province. Sixteen healthcare workers are reported to have been infected.

On 24 January 2020, the first three cases of 2019-nCoV imported into the EU/EEA were identified in France and one additional case was reported on 29 January 2020. On 28 January, a cluster of four locally-acquired cases, with indirect links to Wuhan, was reported from Germany. On 29 January, Finland reported an imported case from Wuhan.

China CDC assesses the transmissibility of this virus to be sufficient for sustained community transmission without unprecedented control measures. Further cases and deaths in China are expected in the coming days and weeks. Further cases or clusters are also expected among travellers from China, mainly Hubei province. Therefore, health authorities in the EU/EEA Member States should remain vigilant and strengthen their capacity to respond to such an event.

There are considerable uncertainties in assessing the risk of this event, due to lack of detailed epidemiological analyses.

On the basis of the information currently available, ECDC considers that:

- the potential impact of 2019-nCoV outbreaks is high;
- the likelihood of infection for EU/EEA citizens residing in or visiting Hubei province is estimated to be high;
- the likelihood of infection for EU/EEA citizens in other Chinese provinces is moderate and will increase;
- there is a moderate-to-high likelihood of additional imported cases in the EU/EEA;
- the likelihood of observing further limited human-to-human transmission within the EU/EEA is estimated as very low to low if cases are detected early and appropriate infection prevention and control (IPC) practices are implemented, particularly in healthcare settings in EU/EEA countries;
- assuming that cases in the EU/EEA are detected in a timely manner and that rigorous IPC measures are applied, the likelihood of sustained human-to-human transmission within the EU/EEA is currently very low to low;
- the late detection of an imported case in an EU/EEA country without the application of appropriate infection prevention and control measures would result in the high likelihood of human-to-human transmission, therefore in such a scenario the risk of secondary transmission in the community setting is estimated to be high.

Source: [4]

## Editorial note

This editorial was published as an e-Alert on 31 January 2020.
